# Blocking ATP-sensitive potassium channel alleviates morphine tolerance by inhibiting HSP70-TLR4-NLRP3-mediated neuroinflammation

**DOI:** 10.1186/s12974-017-0997-0

**Published:** 2017-11-25

**Authors:** Jie Qu, Xue-You Tao, Peng Teng, Yan Zhang, Ci-Liang Guo, Liang Hu, Yan-Ning Qian, Chun-Yi Jiang, Wen-Tao Liu

**Affiliations:** 1 Neuroprotective Drug Discovery Key Laboratory of Nanjing Medical University, Department of Pharmacology, Nanjing Medical University, 101 Longmian Avenue, Nanjing, Jiangsu 211166 China; 2 Department of Anesthesiology, Yangzhou Maternal and Child Health Hospital Affiliated with Yangzhou Medical University, Yangzhou, China; 3 Research Division of Pharmacology, China Pharmaceutical University, Nanjing, China; 4 Department of Anesthesiology, 1st Affiliated Hospital, Nanjing Medical University, Nanjing, China; 5 Department of Pharmacy, Sir Run Run Shaw Hospital Affiliated to Nanjing Medical University, Nanjing, China

**Keywords:** Morphine tolerance, HSP70, TLR4, K_ATP_ channel, Neuroinflammation

## Abstract

**Background:**

Long-term use of morphine induces analgesic tolerance, which limits its clinical efficacy. Evidence indicated morphine-evoked neuroinflammation mediated by toll-like receptor 4 (TLR4) - NOD-like receptor protein 3 (NLRP3) inflammasome was important for morphine tolerance. In our study, we investigated whether other existing alternative pathways caused morphine-induced activation of TLR4 in microglia. We focused on heat shock protein 70 (HSP70), a damage-associated molecular pattern (DAMP), which was released from various cells upon stimulations under the control of K_ATP_ channel and bound with TLR4-inducing inflammation. Glibenclamide, a classic K_ATP_ channel blocker, can improve neuroinflammation by inhibiting the activation of NLRP3 inflammasome. Our present study investigated the effect and possible mechanism of glibenclamide in improving morphine tolerance via its specific inhibition on the release of HSP70 and activation of NLRP3 inflammasome induced by morphine.

**Methods:**

CD-1 mice were used for tail-flick test to evaluate morphine tolerance. The microglial cell line BV-2 and neural cell line SH-SY5Y were used to investigate the pharmacological effects and the mechanism of glibenclamide on morphine-induced neuroinflammation. The activation of microglia was accessed by immunofluorescence staining. Neuroinflammation-related cytokines were measured by western blot and real-time PCR. The level of HSP70 and related signaling pathway were evaluated by western blot and immunofluorescence staining.

**Results:**

Morphine induced the release of HSP70 from neurons. The released HSP70 activated microglia and triggered TLR4-mediated inflammatory response, leading to the phosphorylation of p38 mitogen-activated protein kinase (MAPK) and nuclear factor-κB (NF-κB) p65 and the activation of NLRP3 inflammasome. Moreover, anti-HSP70 neutralizing antibody partly attenuated chronic morphine tolerance. The secretion of HSP70 was under the control of MOR/AKT/K_ATP_/ERK signal pathway. Glibenclamide as a classic K_ATP_ channel blocker markedly inhibited the release of HSP70 induced by morphine and suppressed HSP70-TLR4-NLRP3 inflammasome-mediated neuroinflammation, which consequently attenuated morphine tolerance.

**Conclusions:**

Our study indicated that morphine-induced extracellular HSP70 was an alternative way for the activation of TLR4-NLRP3 in analgesic tolerance. The release of HSP70 was regulated by MOR/AKT/K_ATP_/ERK pathway. Our study suggested a promising target, K_ATP_ channel and a new leading compound, glibenclamide, for treating morphine tolerance.

**Electronic supplementary material:**

The online version of this article (10.1186/s12974-017-0997-0) contains supplementary material, which is available to authorized users.

## Background

For centuries, opioids have been the mainstay of acute and chronic pain treatment. Morphine as a classic opioid analgesic is a golden standard for treating severe pain. However, long-term use of morphine induces analgesic tolerance, which limits its clinical efficacy. The mechanism of morphine tolerance is complicated, which involves many aspects, such as receptors, ion channels, and neuroinflammation [1–3]. Among all the aspects, the activation of microglia was thought to be important in the development and maintenance of morphine tolerance. Chronic morphine administration activated microglia in the spinal cord [4–10], and the activated microglia released proinflammatory factors such as IL-1β, tumor necrosis factor-α (TNF-α), and IL-6 [11] and upregulated the expression of cell-surface receptors, such as TLR4, which participated in the neuroinflammatory process [12–17].

Numerous studies reported that morphine caused the activation of microglia by TLR4 [4, 18], and morphine triggered TLR4-mediated neuroinflammation by binding with MD-2, a TLR4 accessory protein [4]. TLR4 is a pattern-recognition receptor that recognizes specific DAMPs and subsequently initiates immune response [19, 20]. It was also reported that blockade of TLR4 inhibited the activation of microglia and attenuated morphine tolerance [21], which suggested that TLR4 was very important in the development of morphine tolerance. However, the mechanism of TLR4 activation during morphine tolerance is not fully understood.

TLR4 activation triggered the downstream signaling pathway acting as signal 1 for the activation of NLRP3 inflammasome, which was a protein complex for processing the maturation of IL-1β and IL-18 [22, 23]. It was reported that the activation of NLRP3 inflammasome involved a two-step process. The first step, signal 1, primed signals such as DAMPs [24], stimulated TLR4, and then enhanced NF-κB-driven transcription of NLRP3 and pro-IL-1β [25]. Then, a second signal, signal 2, promoted numerous NLRP3 to form a protein complex with apoptosis-associated speck-like protein (ASC) containing a caspase recruitment domain [26, 27] and led to the conversion of pro-IL-1β to IL-1β. NLRP3 inflammasome was crucial in morphine tolerance and inhibition of NLRP3 inflammasome-alleviated morphine tolerance [5, 28].

There are several endogenous TLR4 agonists, such as heat shock proteins (HSPs), high-mobility group box 1 (HMGB1), and debris produced from the digestion of the extracellular matrix (ECM) [29]. Previous studies noted out that HSP70, as an important DAMP, was released from intact human prostate carcinoma cell lines (PC-3 and LNCaP) by a mechanism independent of de novo HSP70 synthesis or cell death [30, 31]. The release of HSP70 was regulated by the ATP-binding cassette (ABC) family transporter proteins [32]. Studies indicated that morphine could activate K_ATP_ channel [33], which is an ABC transporter [34]. Moreover, it was reported that the opening of the K_ATP_ channel could upregulate HSP70 expression [35]. Hence, we want to investigate whether morphine could induce the release of HSP70 by activating K_ATP_ channel.

Therefore, we focused on the role of HSP70, an abundant and quickly inducible protein that was constitutively expressed at normal growth temperatures and functions as a molecular chaperone in the life cycle of proteins, in morphine-induced neuroinflammation [36–38]. Moreover, HSP70 can be released rapidly upon the stimulation from various cells [39, 40], and the extracellular HSP70 could induce inflammatory mediators in a TLR4 dependent manner [41].

Based on the information mentioned above, morphine-evoked TLR4-NLRP3 inflammasome-mediated neuroinflammation was important for morphine tolerance, and HSP70 as an endogenous TLR4 ligand was released immediately upon the stimulation from various cells. Hence, we make the hypothesis that morphine activates TLR4 by inducing the release of HSP70, thereby inducing the activation of NLRP3 inflammasome and leading to neuroinflammation. In our study, we investigated the mechanism of morphine-induced HSP70 release and the effect of HSP70-TLR4-NLRP3 inflammasome-mediated neuroinflammation in morphine tolerance.

## Methods

### Animals

Adult male CD-1 mice (18–22 g) were provided by the Experimental Animal Center at Nanjing Medical University, Nanjing, China. Animals were housed five to six per cage under pathogen-free conditions with soft bedding under controlled temperature (22 ± 2 °C) and a 12-h light/dark cycle (lights on at 8:00 a.m.). Behavioral testing was performed during the light cycle (between 9:00 a.m. and 5:00 p.m.). The animals were allowed to acclimate to these conditions for at least 2 days before starting experiments. For each group of experiments, the animals were matched by age and body weight.

### Chemicals and reagents

Glibenclamide, TAK242, lipopolysaccharide (LPS), dimethyl sulfoxide (DMSO), and ATP were purchased from Sigma-Aldrich (St. Louis, MO, USA). Morphine hydrochloride was purchased from Shenyang First Pharmaceutical Factory, Northeast Pharmaceutical Group Company (Shenyang, China). Kir6.2 siRNA and control siRNA were purchased from GenePharma (Shanghai, China). SCH77298 and SB202190 were purchased from MedChemExpress (NJ, USA). AZD5363 and naloxone were purchased from Selleckchem (Houston, TX, USA). Dil (an orange fluorescent membrane dye) was purchased from KenGEN (KenGEN BioTECH, China). Antibody for β-actin was from Sigma-Aldrich (St. Louis, MO, USA). Antibodies for heat shock protein 70 (HSP70), p38, phosphorylated p38 (Tyr182), phospho-NF-κB p65 (Ser536), ERK, phosphorylated ERK (Thr202/Tyr204), AKT, and phosphorylated AKT (Ser473) were from Cell Signaling Technology (Beverly, MA, USA). Antibodies for NLRP3 and caspase-1 were from Adipogen International (San Diego, CA, USA). Antibody for IL-1β was purchased from R&D Systems (Minneapolis, MN, USA). Antibody for Kir6.2 was purchased from Santa Cruz Biotechnology (Santa Cruz, CA, USA). Secondary antibodies for western blot were from Sigma-Aldrich (St. Louis, MO, USA). Immunofluorescent antibodies for Iba-1 and HSP70 were from Abcam (Cambridge, MA, USA). Immunofluorescent antibodies for c-fos and CGRP were from Cell Signaling Technology (Beverly, MA, USA). Secondary antibodies for immunofluorescence were from Jackson Immunoresearch Laboratories (West Grove, PA, USA) and Abcam (Cambridge, MA, USA). Normal mouse IgM and anti-HSP70 mouse monoclonal IgM were from Santa Cruz Biotechnology (Santa Cruz, CA, USA). Fetal bovine serum (FBS) was purchased from Gibco, and other cell culture media and supplements were purchased from KenGEN (KenGEN BioTECH, China).

### Tolerance model and behavioral analysis

Animals were habituated in the testing environments for 2 days and carried out behavioral testing in a blinded manner. Glibenclamide was suspended with 1 μg/μL morphine. For the test of chronic tolerance, mice were intrathecally injected with vehicle or morphine (10 μg/10 μL) once daily for seven consecutive days with or without glibenclamide (0.08, 0.4, or 2 μg/10 μL). Behavioral testing was performed 1 h after morphine administration by tail-flick assay every morning. Briefly, mice’s tails were placed in 52 °C water, and the latency of tail withdrawal was measured. A cut-off time of 10 s was set to avoid tissue damage.

### Intrathecal injection procedure

To perform intrathecal (i.t.) injections, the mice were placed in a prone position and the midpoint between the tips of the iliac crest was located. A Hamilton syringe with 30-gauge needle was inserted into the subarachnoid space of the spinal cord between the L5 and L6 spinous processes. Proper intrathecal injection was systemically confirmed by observation of a tail flick. Intrathecal injection did not affect baseline responses, compared with latencies recorded before injection.

### Cell cultures

BV-2 cells were maintained in humidified 5% CO_2_ at 37 °C in Dulbecco’s Modified Eagle’s Medium (DMEM, KenGEN BioTECH, China) supplemented with 10% (*v*/*v*) FBS (Gibco), 80 U/mL penicillin, and 0.08 mg/mL streptomycin, and SH-SY5Y cells were maintained in humidified 5% CO_2_ at 37 °C in Modified Eagle Media: F-12 (MEM/F12, KenGEN BioTECH, China) supplemented with 10% (*v*/*v*) FBS (Gibco), 80 U/mL penicillin and 0.08 mg/mL streptomycin. For further experiments, 10^5^ cells were plated in 6-well plate or 12-well plate overnight and then treated with morphine (200 μM) in the following morning with or without glibenclamide for 12 h. Cell extracts and precipitated supernatants were analyzed by immunoblot assay or real-time PCR.

### Cell immunofluorescence assay

SH-SY5Y cells were plated in glass bottom cell culture dishes and treated with morphine (100, 200, 400 μM) for 12 h with or without glibenclamide (200 μM). Then, SH-SY5Y cells were fixed with 4% paraformaldehyde and were permeated with 0.3% Triton X-100. After blocking with 10% donkey serum in phosphate-buffered saline (PBS) for 2 h, the coverslips with SH-SY5Y cells were incubated at 4 °C with the HSP70 antibody (1:50) or Kir6.2 antibody (1:50) diluted in PBS overnight. Then, the coverslips were exposed to secondary antibodies (1:100, at room temperature for 1 h) and then were rinsed three times with PBS. 4′,6-Diamidino-2-phenylindole (DAPI) is a fluorescent DNA dye to mark nucleus and Dil is an orange fluorescent membrane dye to mark cell membrane. Confocal microscopy analyze was carried out using Carl Zeiss LSM710 confocal system.

### Western blot

Samples (cells or spinal cord tissue segments at L1–L6) were collected and washed with ice-cold PBS before being lysed in radio immunoprecipitation assay (RIPA) lysis buffer, and then sample lysates were separated by SDS-PAGE and electrophoretically transferred onto polyvinylidene fluoride membranes (Millipore). The membranes were blocked with 10% milk in TBST (Tris–HCl, NaCl, Tween 20) for 2 h at room temperature and then probed with primary antibodies at 4 °C for overnight. Finally, the horseradish peroxidase (HRP)-coupled secondary antibodies were utilized for detecting corresponding primary antibody. The primary antibodies utilized included β-actin (1:5000), p38 (1:1000), p-p38 (Tyr182) (1:1000), caspase-1 (1:1000), IL-1β (1:300), HSP70 (1:1000), p-p65 (Ser536) (1:1000), ERK (1:1000), p-ERK (Thr202/Tyr204) (1:1000), AKT (1:1000), p-AKT (Ser473) (1:1000), NLRP3 (1:1000) and caspase-1 (1:1000), and Kir6.2 (1:500). The bands were then developed by enhanced chemiluminescence reagents (PerkinElmer, Waltham, MA, USA). Data were analyzed with the Molecular Imager and the associated software Quantity One-4.6.5 (Bio-Rad Laboratories, USA).

### Immunohistochemistry

Under deep anesthesia by intraperitoneal injection of chloral hydrate (400 mg/kg), animals were perfused with normal saline followed by 4% paraformaldehyde in 0.1 M PBS, pH 7.2–7.4, for 20 min. Then, L4 and L5 lumbar segments were dissected out and post-fixed in the same fixative. The embedded blocks were sectioned as 25 μm thick and processed for immunofluorescence assay. Sections from each group (four mice in each group) were incubated with primary antibodies: Iba-1 (1:200), c-fos (1:200), and CGRP (1:200). Then, the free-floating sections were washed with PBS and incubated with the secondary antibodies (1:300) for 2 h at room temperature. After being washed three times with PBS, the samples were investigated with an immunofluorescence microscope (Zeiss AX10, Germany). Images were randomly coded, and the fluorescence intensities of Iba-1, CGRP, and c-fos positive dots were analyzed by ImageJ software. The average fluorescence intensity of each pixel was normalized to the background intensity in the same image.

### RNA interference

Kir6.2 siRNA and control siRNA were purchased from GenePharma (Shanghai, China). The sequence of Kir6.2 siRNA is as follows: sense: CCAAGCCCAAGUUCAGCAUTT and antisense: AUGCUGAACUUGGGCUUGGTT. Control siRNA was used as a negative control. For the transfection of siRNA, SH-SY5Y cells were cultured in 6-well plates with antibiotic-free medium the day before transfection. The transfection was conducted when cells reached 50 ~ 70% confluence using Lipofectamine 2000 (Invitrogen, USA) and serum-free medium according to the manufacturer’s instructions. After 4 h, the transfection medium was replaced with the culture medium containing 10% FBS and then incubated at 37 °C.

### Quantitative PCR

Total RNA was extracted from BV-2 cells using Trizol reagent (Invitrogen, CA, USA). Isolated RNA was reverse transcribed into cDNA using Prime-Script™ RT Reagent Kit (TaKaRa, Japan) following standard protocol. Quantitative real-time PCR (qPCR) was performed with synthetic primers and SYBR Green (TaKaRa, Japan) with QuantStudio 5 Real-Time PCR Detection System (Thermo Fisher scientific). The relative expression levels of *Il1b* and *Tnfa* were calculated and quantified with the 2^−ΔΔCt^ method after normalization with reference. All primers used are listed in Table 1.

**Table 1:** Sequences of primers for real-time quantitative polymerase chain reaction

Gene		Sequence
*Gapdh*	Forward	5′-GGCATGGACTGTGGTCATGAG-3′
	Reverse	5′-TGCACCACCAACTGCTTAGC-3′
*Illb*	Forward	5′-TCATTGTGGCTGTGGAGAAG-3’
	Reverse	5′-AGGCCACAGGTATTTTGTCG-3’
*Tnfa*	Forward	5′-CATCTTCTCAAAATTCGAGTGACAA-3’
	Reverse	5′-TGGGAGTAGACAAGGTACAACCC-3’

*Gapdh* glyceraldehyde 3-phoshate dehydrogenase, *Illb* interleukin-1β, *Tnfa* tumor necrosis factor-α

### Collection of cerebrospinal fluid (CSF)

Adult male Sprague-Dawley rats (200–250 g) were housed under a 12-h light/dark cycle, with food and water available ad lib. The animals were done under anesthesia induced by chloral hydrate (300 mg/kg, i.p.). The CSF was carefully collected from the cisterna magna of each rat, as described previously, and inspected for blood contamination. Contaminated samples were discarded. Approximately 80 μL of CSF was collected from each animal. After a short centrifugation step (5 min at 5000*g*, 4 °C), the samples were dissolved in 2× Laemmli SDS loading buffer, boiled, and analyzed by SDS-PAGE followed by western blotting.

### Statistical analysis

GraphPad Prism 6 software (GraphPad Software, San Diego, CA, USA) was used to conduct all the statistical analyses. The differences between two groups were evaluated by Student’s *t* test. The data from more than two groups were evaluated by one-way ANOVA or two-way ANOVA. Results were represented as mean ± SEM of the independent experiments. Results described as significant were based on a criterion of *P* < 0.05.

## Results

### Morphine induces the release of HSP70 from neurons by activating K_ATP_ channel

**Fig. 1:**
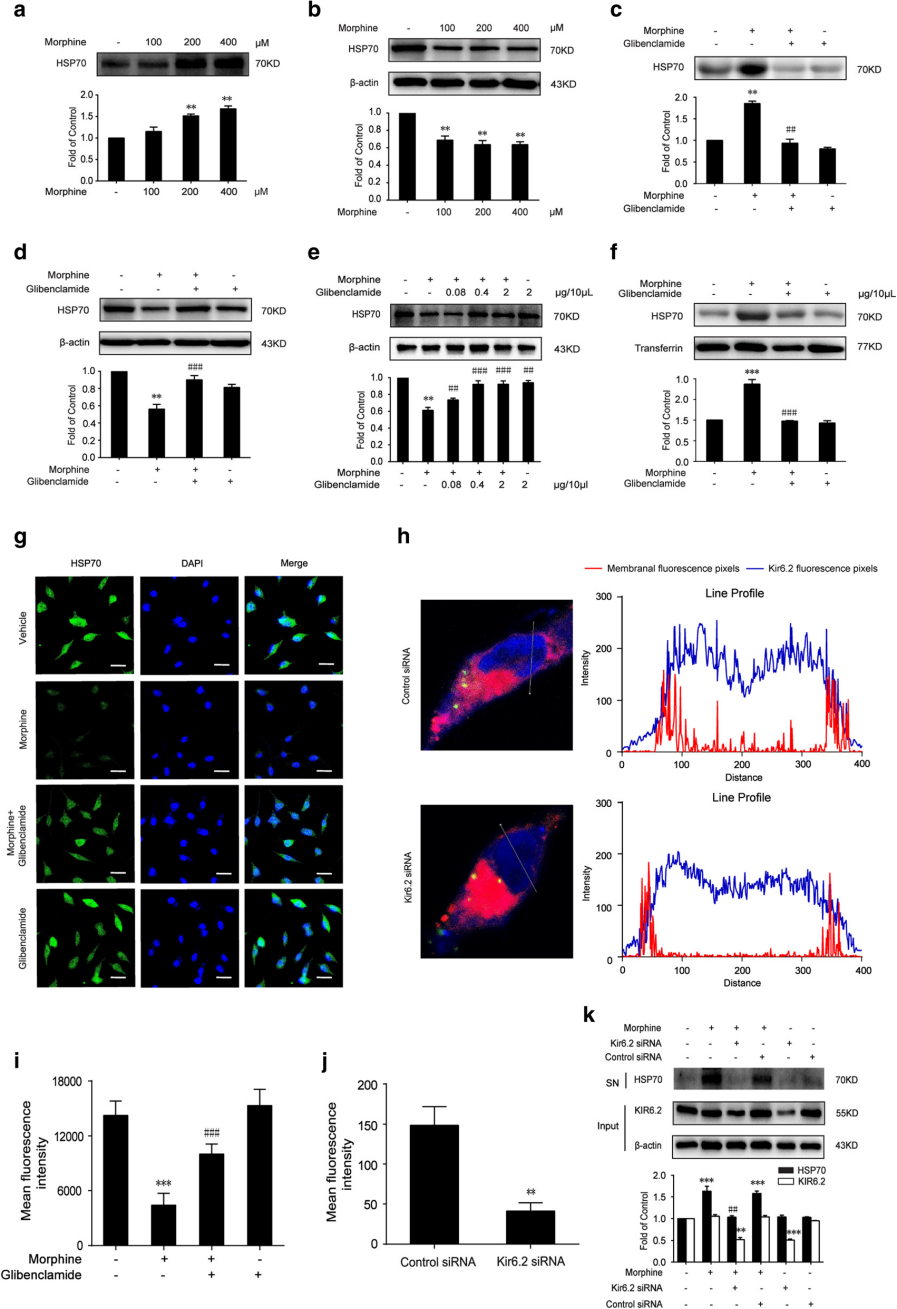
Morphine induces the release of HSP70 from neurons by activating K_ATP_ channel **a** morphine induced the efflux of HSP70 into extracellular environment in a concentration-dependent manner in SH-SY5Y cells. Supernatants were collected 12 h after morphine (200 μM) and analyzed by western blot (*n* = 3). **b** morphine decreased the intracellular protein level of HSP70 in SH-SY5Y cells. Cell extracts were collected 12 h after morphine (200 μM) treatment and analyzed by immunoblot assay (*n* = 3). **c** Glibenclamide administration (200 μM, 15 min) prior to morphine (200 μΜ, 12 h) prevented the morphine-induced HSP70 release in SH-SY5Y cells. Supernatants were collected 12 h after morphine treatment and determined by western blot (*n* = 3). **d** Glibenclamide (200 μM) inhibited the decrease of intracellular HSP70 caused by morphine in SH-SY5Y cells. Cell extracts were collected 12 h after morphine treatment and analyzed by immunoblot assay (*n* = 3). **e** Consecutive administration of glibenclamide (0.4 and 2 μg/10 μL, i.t.) for 7 days inhibited the decrease of HSP70 induced by morphine (10 μg/10 μL, i.t.) in the spinal cord. The spinal samples were collected 1 h after the last morphine treated and determined by western blot (*n* = 4). **f** Consecutive administration of glibenclamide (2 μg/10 μL, i.t.) for 7 days inhibited the release of HSP70 induced by morphine (10 μg/10 μL, i.t.) in CSF. CSF was collected from rats 1 h after the last administration and determined by western blot (*n* = 4). Transferrin is used as a loading control. **g**, **i** Glibenclamide administration (200 μM, 15 min) prior to morphine (200 μΜ, 12 h) inhibited the release of HSP70 from SH-SY5Y cells induced by morphine. The green fluorescence indicated the intracellular level of HSP70 in SH-SY5Y cells (*n* = 4). Scale bar, 75 μm. **h**, **j** Kir6.2 siRNA downregulated the level of Kir6.2 on the cell membrane of SH-SY5Y cells. Kir6.2 siRNA or control siRNA was transfected to SH-SY5Y cells. Blue fluorescence labeled Kir6.2, red fluorescence labeled cell membrane, and green fluorescence indicated that FAM-labeled siRNA was successfully transfected into SH-SY5Y cells. The blue curves represent the line profile of Kir6.2, and the red curves represent the line profile of cell membrane. The fluorescence intensity of blue curve overlapped with red curve was utilized to represent the level of Kir 6.2 distributed on cell membrane. The data was obtained from four independent experiments (*n* = 4). Scale bar 10 μm. **k** Knockdown of Kir6.2 abolished morphine-induced release of HSP70. SH-SY5Y cells were transfected with Kir6.2 siRNA or control siRNA for 18 h, followed by 200 μM morphine treatment for 12 h. The efficiency of Kir6.2 knockdown was assessed by immunoblot assay (*n* = 3). **a**–**k** Data were analyzed by one-way ANOVA. **h** Data were analyzed by Student’s *t* test **P < 0.01, ***P < 0.001 vs. vehicle, ^##^ *P* < 0.01, ^###^ *P* < 0.001 vs. the morphine-treated group

We utilized neural cell line SH-SY5Y cells to investigate whether morphine could induce the efflux of HSP70 into extracellular environment. We incubated SH-SY5Y cells with different concentrations (100, 200, 400 μM) of morphine for 12 h, then the supernatants of SH-SY5Y cells were collected and analyzed by western blot. MTT assay indicated that the different concentrations (100, 200, 400 μM) of morphine did not affect cell proliferation (Additional file 1: Figure S1). We found that morphine promoted the efflux of HSP70 into extracellular environment in a concentration-dependent manner (Fig. 1a); accordingly, morphine decreased HSP70 protein level in SH-SY5Y cells (Fig. 1b). Furthermore, we also investigated the effect of morphine on microglia besides that on neurons. BV-2 cells were utilized, and the immunoblot result showed that morphine did not induce the release of HSP70 from microglia (Additional file 2: Figure S2).

A previous study pointed out that the release of HSP70 was regulated by the ABC family transporter proteins [32], and morphine could activate K_ATP_ channel [33], which is an ABC transporter. Hence, we utilized glibenclamide, a classic K_ATP_ channel blocker, to study the role of K_ATP_ channel in the release of HSP70 induced by morphine. We found that glibenclamide decreased the extracellular HSP70 (Fig. 1c) and increased intracellular HSP70 upon morphine stimulation in SH-SY5Y cells (Fig. 1d). In vivo study, we found that morphine decreased the level of intracellular HSP70 in the spinal cord (Fig. 1e). To further confirm the release of HSP70 induced by morphine, we established morphine tolerance model with SD rats to collect the CSF (Additional file 3: Figure S3). Immunoblot data indicated that morphine markedly caused HSP70 released into CSF (Fig. 1f). Moreover, glibenclamide significantly inhibited the efflux of HSP70 induced by morphine. It suppressed the decline of intracellular HSP70 (Fig. 1e) and deceased the level of HSP70 in CSF (Fig. 1f) during morphine tolerance.

In order to further investigate whether morphine-induced release of HSP70 was K_ATP_ channel dependent, we utilized Kir6.2 small interfering RNA to downregulate Kir6.2. Kir6.2 is the pore-forming subunit of K_ATP_ channel, which is predominantly expressed on neurons. We found that Kir6.2 siRNA downregulated the level of Kir6.2 on cell membrane of SH-SY5Y cells (Fig. 1h, j), and knockdown of Kir6.2 abolished morphine-induced HSP70 release (Fig. 1k). It demonstrated that K_ATP_ channel was essential for the morphine-induced HSP70 release. These data provided sufficient evidences that morphine induced the release of HSP70 by activating K_ATP_ channel.

Moreover, we found that glibenclamide inhibited the release of HSP70 from SH-SY5Y cells induced by morphine, and the immunofluorescence intensity of HSP70 increased in the presence of glibenclamide compared with only morphine group (Fig. 1g, i).

### Extracellular HSP70 triggers inflammatory response dependent on TLR4 in microglia

**Fig. 2:**
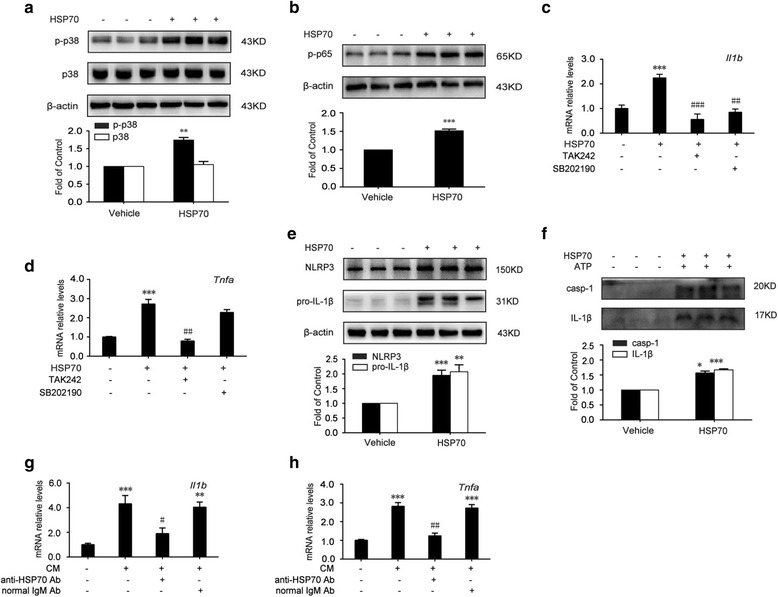
Extracellular HSP70 triggers inflammatory response dependent on TLR4 in microglia. **a**, **b** Recombinant mouse HSP70 (100 ng/mL, 12 h) upregulated the phosphorylation of p38 MAPK and NF-κB p65 in BV-2 cells. Cell extracts were collected and analyzed by western blot (*n* = 3). **c**, **d** The levels of *Il1b* and *Tnfa* mRNAs in response to HSP70 under treatment of TLR4 antagonist or p38 inhibitor were assessed in BV-2 cells. Cells were pretreated with TLR4 antagonist (TAK242, 10 μM) or p38 inhibitor (SB202190, 10 μM) for 15 min, followed by recombinant mouse HSP70 (100 ng/mL) treatment. Then, cell extracts were collected 12 h after HSP70 treatment and analyzed by qPCR (*n* = 3). **e** Recombinant mouse HSP70 (100 ng/mL, 12 h) increased the levels of pro-IL-1β and NLRP3 in BV-2 cells. Cell extracts were collected and analyzed by western blot (*n* = 3). **f** BV-2 cells were stimulated by recombinant mouse HSP70 (100 ng/mL) for 12 h, and then, the inflammasome was activated with 5 mM of ATP for 0.5 h, inducing the maturation of caspase-1 and IL-1β. Supernatants of BV-2 cells were collected and analyzed by western blot (*n* = 3). **g**, **h** Conditional medium collected from morphine-treated (200 μM, 12 h) SH-SY5Y cells incubated BV-2 cells for 12 h in presence of anti-HSP70 antibody (100 ng/mL) or normal IgM (100 ng/mL), and then, the cell extracts were collected and analyzed by qPCR (*n* = 3). **a**, **b**, **e**, and **f** Data were analyzed by Student’s *t* test. **c**, **d**, **g**, and **h** Data were analyzed by one-way ANOVA.*P < 0.05, **P < 0.01, ***P < 0.001 vs. vehicle, ^##^ *P* < 0.01, ^###^ *P* < 0.001 vs. the HSP70-treated group

Next, we investigated the role of extracellular HSP70 in neuroinflammation. Microglia, predominant TLR4 expressed cells [29, 42], are the innate immune cells in central nervous system. Once activated, they released proinflammatory cytokines, including TNF-α and IL-1β [43–45]. TLR4 can be activated by DAMPs, triggering its downstream signaling pathway, consequently evoking innate immune response through the maturation of IL-1β [46, 47]. As HSP70 is an important endogenous TLR4 agonist, we investigated the effect of extracellular HSP70 on inflammatory response and immortalized murine microglial cell line BV-2 was utilized [48, 49]. Recombinant mouse HSP70 (100 ng/mL) significantly upregulated the phosphorylation of p38 MAPK and NF-κB p65 in BV-2 cells (Fig. 2a, b). In accordance with above, extracellular HSP70 increased the transcription of IL-1β and TNF-α (Fig. 2c, d). Furthermore, the administration of TLR4 antagonist (TAK242, 10 μM) suppressed the upregulation of the proinflammatory cytokine transcription in HSP70-treated cells (Fig. 2c, d). These results indicated that TLR4 was essential for inflammatory response caused by HSP70. However, we found that treatment of p38 inhibitor (SB202190, 10 μM) only suppressed the upregulation of IL-1β mRNAs but not TNF-α in HSP70-treated cells (Fig. 2c, d). The administration of recombinant mouse HSP70 increased the protein level of NLRP3 and pro-IL-1β (Fig. 2e), and in the presence of ATP, the priming HSP70 increased the activation of caspase-1 and the maturation of IL-1β compared with the vehicle group (Fig. 2f).

In order to further confirm the role of HSP70 in inducing inflammatory response, we utilized conditional medium (CM) from morphine-treated (200 μM, 12 h) SH-SY5Y cells to activate BV-2 cells. Then, we found CM increased the transcription of IL-1β and TNF-α mRNA. Furthermore, anti-HSP70 antibody (100 ng/mL) suppressed CM-induced upregulation of IL-1β and TNF-α, and normal IgM (100 ng/mL) did not show an inhibitory effect (Fig. 2g, h). Therefore, our findings indicated that HSP70 could act as a priming signal to cause TLR4-dependent inflammatory response, and HSP70 is very important for morphine-induced neuroinflammation.

### Glibenclamide attenuates morphine tolerance and suppresses morphine-induced microglia activation

According to the abovementioned, morphine induced the release of HS70 and extracellular HSP70-caused inflammatory response in microglia. We questioned whether the release of HSP70 was significant for the development of morphine tolerance.

**Fig. 3:**
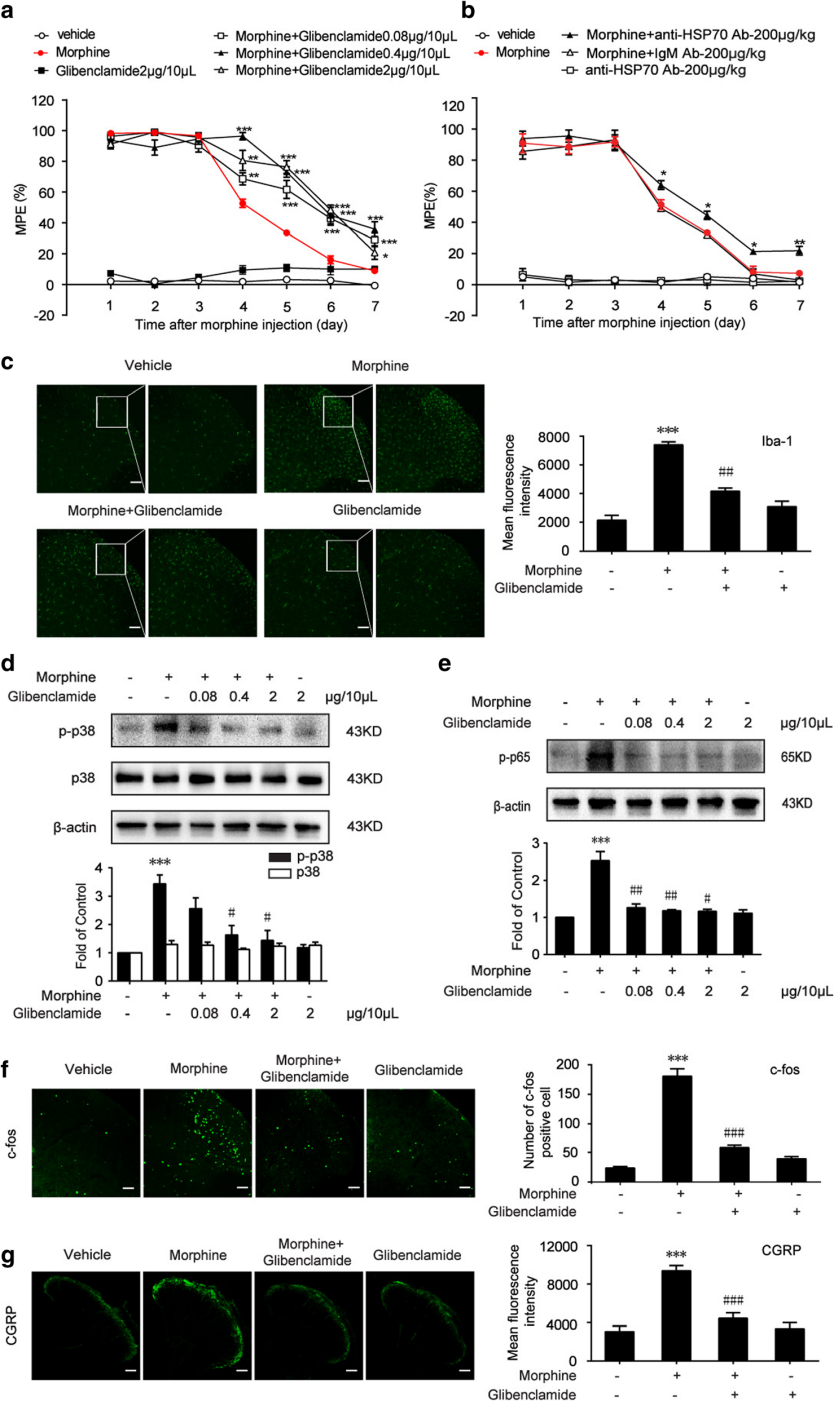
Glibenclamide attenuates morphine tolerance and suppresses morphine-induced microglia activation. Tail-flick method was performed to evaluate the effect of glibenclamide on the morphine tolerance. Data were shown as percentage of maximal possible effect (MPE). **a** Glibenclamide co-administration with morphine improved chronic morphine tolerance in mice (*n* = 8). Morphine (10 μg/10 μL) was intrathecally injected with different doses of glibenclamide (0.08, 0.4, and 2 μg/10 μL) once daily, and the MPE was measured 1 h after the first injection of each day. **b** Consecutive administration of anti-HSP70 neutralizing antibody (200 μg/kg, i.t.) once daily, partially attenuating morphine tolerance in mice (*n* = 6). **c** Immunofluorescence result showed that glibenclamide (2 μg/10 μL) significantly inhibited the activation of microglia evoked by morphine in the spinal cord (*n* = 4). **d**, **e** Immunoblot results demonstrated that glibenclamide (0.08, 0.4, and 2 μg/10 μL) suppressed morphine-induced upregulation of phosphorylation of p38 MAPK and NF-κB p65, but not the p38 total protein in the spinal cord. (*n* = 4). **f**, **g** Immunofluorescence analysis showed that glibenclamide (2 μg/10 μL) markedly inhibited the activation of neuronal c-fos and CGRP after morphine treatment in the spinal cord. The quantification of c-fos and CGRP immunofluorescence was respectively represented as number of c-fos-positive cells and mean fluorescence intensity of CGRP in dorsal horn (*n* = 4). Glibenclamide (0.08, 0.4, and 2 μg/10 μL) was administered once daily for 7 days. One hour after the final administration, spinal samples were collected. **a**, **b** data were analyzed by two-way ANOVA. **c**-**g** data were analyzed by one-way ANOVA. *P < 0.05, **P < 0.01, ***P < 0.001 vs. vehicle, ^#^ *P* < 0.05, ^##^ *P* < 0.01, ^###^ *P* < 0.001 vs. morphine-treated group. Scale bar: 75 μm

Glibenclamide and anti-HSP70 neutralizing antibody were utilized to investigate the therapeutic effects in morphine tolerance. The behavioral test results showed that glibenclamide attenuated morphine tolerance in a dose-dependent manner (Fig. 3a), and functional antagonism of extracellular HSP70 with anti-HSP70 neutralizing antibody (200 μg/kg) partially attenuated morphine tolerance (Fig. 3b). The MPE decreased to 8.88% in chronically morphine-treated mice on day 7. The reduction in morphine’s MPE was significantly prevented by once daily administration of glibenclamide (0.08, 0.4, or 2 μg/10 μL, i.t.) with morphine. Moreover, glibenclamide and anti-HSP70 neutralizing antibody did not affect acute morphine analgesic effect (Additional files 4 and 5: Figures S4 and S5), and glibenclamide (2 μg/10 μL) did not affect the blood glucose threshold after 1 h of its administration compared with vehicle group (Additional file 6: Figure S6).

Microglia has been shown to play a main role in cytokine release upon activation in the CNS [50]. Compelling evidences have suggested that the activation of microglia is crucial for the development of morphine tolerance [51]. Repeated morphine administration led to the release of proinflammatory cytokines TNF-α and IL-1β from microglia [52]. Immunofluorescence data showed that repeated morphine treatment (10 μg/10 μL, once daily for 7 days) led to the activation of microglia (Iba-1 as microglia marker), and glibenclamide (2 μg/10 μL, i.t.) significantly inhibited morphine-induced activation of microglia (Fig. 3c). The activation of microglia induced by morphine showed elongated cell somata with fewer branches but thicker processes, and this kind of morphology is known as the activated morphology [53]. Furthermore, morphine increased the number of microglia. Glibenclamide significantly attenuated this change, the morphology of microglia recovered to rest state and the number decreased compared with morphine group. Glibenclamide also inhibited the phosphorylation of p38 MAPK and NF-κB p65 induced by morphine exposure in the spinal cord (Fig. 3d, e). Furthermore, the expression of c-fos and CGRP protein, two of the immediate early genes rapidly expressed in neurons after a noxious stimulus, were increased in the dorsal horn of the spinal cord after morphine tolerance, and these increases were then suppressed by glibenclamide (Fig. 3f, g).

### Glibenclamide suppresses morphine-induced NLRP3 inflammasome activation

**Fig. 4:**
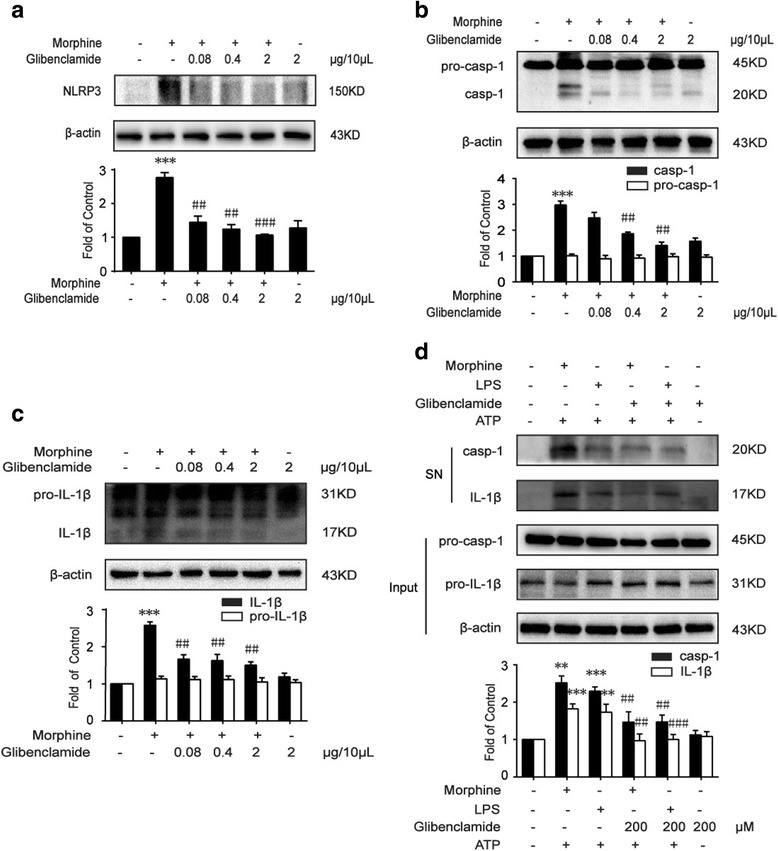
Glibenclamide suppresses morphine-induced NLRP3 inflammasome activation. **a** Glibenclamide (0.08, 0.4, and 2 μg/10 μL) inhibited the morphine-induced upregulation of NLRP3 in the spinal cord (*n* = 4). **b** Glibenclamide (0.4 and 2 μg/10 μL) suppressed the morphine-induced activation of caspase-1 in the spinal cord (*n* = 4). **c** Immunoblot results showed that glibenclamide (0.4 and 2 μg/10 μL) decreased the level of mature IL-1β evoked by morphine in the spinal cord (*n* = 4). Glibenclamide (0.08, 0.4, and 2 μg/10 μL) was administered once daily for 7 days. One hour after the final administration, spinal samples were collected. **d** Glibenclamide (200 μM) inhibited the morphine (200 μM) or LPS (1 μg/mL) induced increase of caspase-1 and IL-1β in BV-2 cells. Morphine or LPS-primed BV-2 cells were incubated for 12 h with glibenclamide for 15 min before ATP (5 mM) was added for another 30 min. Supernatants of BV-2 cells were collected and analyzed by western blot (*n* = 3). Data were analyzed by one-way ANOVA. **P < 0.01, ***P < 0.001 vs. vehicle, ^##^ *P* < 0.01, ^###^ *P* < 0.001 vs. the morphine-treated group

It was reported that NLRP3 inflammasome was crucial in morphine tolerance and inhibition of NLRP3 inflammasome alleviated morphine tolerance [5, 28]. Hence, we tested the effect of glibenclamide on the activation of NLRP3 inflammasome in morphine tolerance. Our data showed that consistent morphine exposure (10 μg/10 μL, once daily for 7 days, i.t.) increased the protein level of NLRP3, caspase-1, and proinflammatory cytokine, IL-1β, in mice spinal cord. The repetitive administration of glibenclamide (0.08, 0.4, or 2 μg/10 μL, i.t.) suppressed the upregulation of these proteins caused by morphine (Fig. 4a–c). Furthermore, in vitro studies, we found that glibenclamide inhibited caspase-1 activation and the secretion of IL-1β induced by morphine or LPS in the presence of ATP (Fig. 4d), the results were consistent with what in vivo, suggesting that glibenclamide could inhibit morphine-induced activation of NLRP3 inflammasome.

### The release of HSP70 induced by morphine depends on MOR/AKT/K_ATP_/ERK pathway

**Fig. 5:**
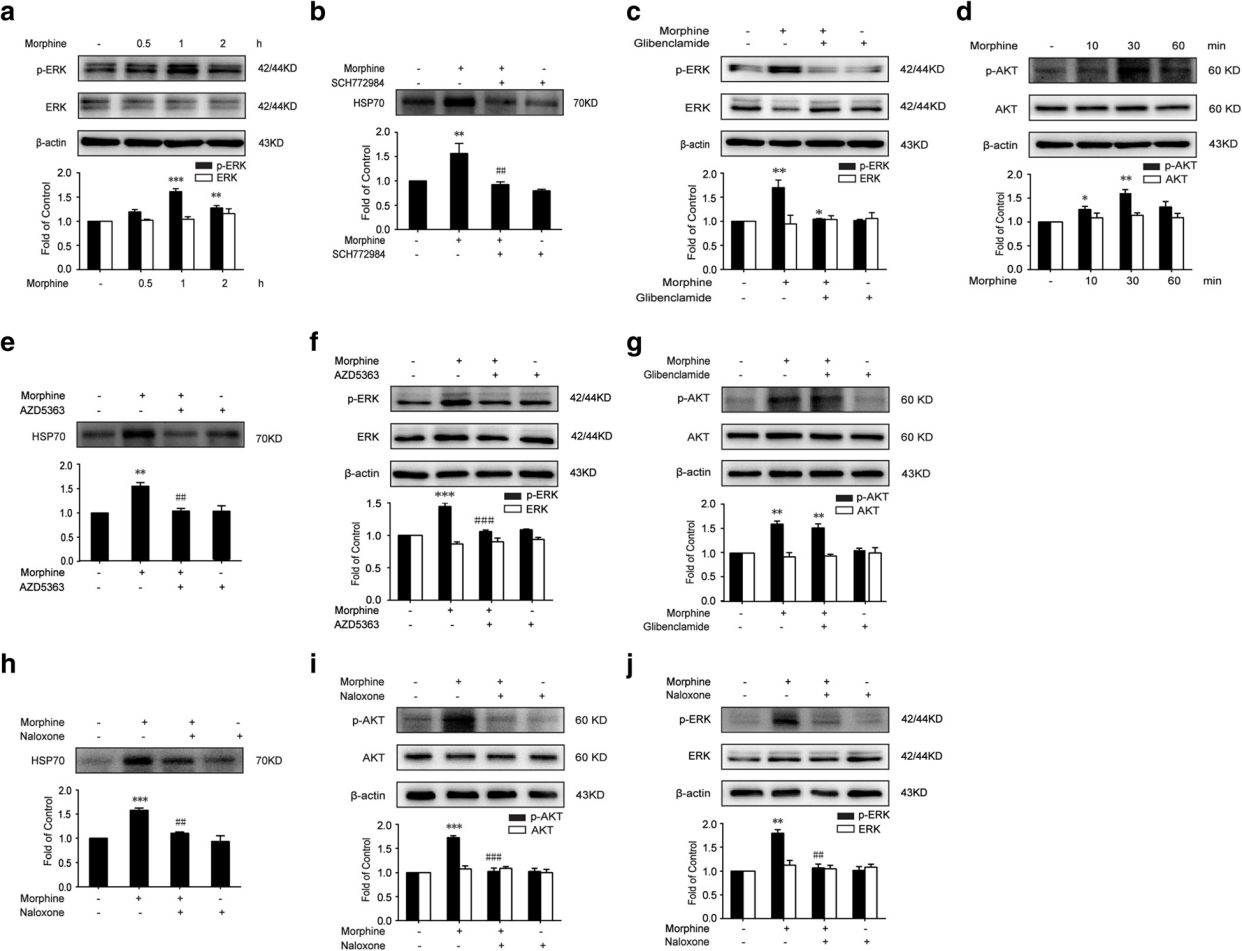
The release of HSP70 induced by morphine depends on MOR/AKT/K_ATP_/ERK pathway. **a** Morphine (200 μM) elevated ERK1/2 phosphorylation in SH-SY5Y cells. Cell extracts were collected and analyzed by western blot (*n* = 3). **b** HSP70 release induced by morphine was reversed by ERK1/2 inhibitor in SH-SY5Y cells. SCH772984 (ERK1/2 inhibitor, 2 μM) was given 15 min before morphine (200 μM, 12 h) administration. Supernatants were collected and analyzed by western blot (*n* = 3). **c** Glibenclamide abolished the phosphorylation of ERK1/2 elevated by morphine. Glibenclamide (200 μM) was given 15 min before morphine (200 μM, 1 h) administration. Cell extracts were collected and analyzed by western blot (*n* = 3). **d** Morphine (200 μM) elevated AKT phosphorylation in SH-SY5Y cells. Cell extracts were collected and analyzed by western blot (*n* = 3). **e** HSP70 release induced by morphine was abrogated by AKT inhibitor. AZD5363 (AKT inhibitor, 2 μM) was given 15 min before morphine (200 μM, 12 h). Supernatants were collected and analyzed 12 h after morphine exposure (*n* = 3). **f** AKT inhibitor suppressed ERK1/2 phosphorylation elevated by morphine in SH-SY5Y cells. AZD5363 (AKT inhibitor, 2 μM) was given 15 min before morphine (200 μM, 1 h) administration. Cell extracts were collected and analyzed by western blot (*n* = 3). **g** Glibenclamide did not affect AKT phosphorylation induced by morphine in SH-SY5Y cells. Glibenclamide (200 μM) was given 15 min before morphine (200 μM, 0.5 h) administration. Cell extracts were collected and analyzed by western blot (*n* = 3). **h** Naloxone inhibited the efflux of HSP70 into extracellular environment promoted by morphine. Naloxone (10 μM) was given 15 min before morphine (200 μM, 12 h) administration. Supernatants were collected and analyzed by western blot (*n* = 3). **i** Naloxone reversed AKT phosphorylation elevated by morphine in SH-SY5Y cells. Naloxone (10 μM) was given 15 min before morphine (200 μM, 0.5 h) administration. Cell extracts were collected and analyzed by western blot (*n* = 3). **j** Naloxone suppressed ERK1/2 phosphorylation induced by morphine in SH-SY5Y cells. Naloxone (10 μM) was given 15 min before morphine (200 μM, 1 h) administration. Cell extracts were collected and analyzed by western blot (*n* = 3). Data were analyzed by one-way ANOVA. *P < 0.05, **P < 0.01, ***P < 0.001 vs. vehicle, ^#^ *P* < 0.05, ^##^ *P* < 0.01, ^###^ *P* < 0.001 vs. the morphine-treated group

The next question addresses how morphine induced the release of HSP70 by K_ATP_ channel. Previous studies indicated that stress-induced secretion of HSP70 was mediated by ERK1/2 [54]. Therefore, we investigated whether morphine-induced release of HSP70 was related to ERK1/2. Our data showed that phosphorylation of ERK1/2 was significantly elevated in SH-SY5Y cells 60 min after morphine (200 μM) administration (Fig. 5a) and SCH772984 (an ERK1/2 inhibitor, 2 μM) for 15 min before morphine administration, significantly inhibited the morphine-induced release of HSP70 (Fig. 5b). One study reported that H_2_S exerted a protective effect against cerebral hypoxia-induced neuronal cell death via K_ATP_/PKC/ERK1/2/HSP90 pathway [55], and they proved that glibenclamide abolished the effect of NaHS on ERK1/2 phosphorylation in SH-SY5Y cells, indicating that ERK1/2 activation was downstream to K_ATP_ channel; thus, we investigated whether or not glibenclamide could inhibit elevated ERK1/2 phosphorylation induced by morphine. Then, we found that glibenclamide (200 μM) preincubation (15 min) prevented ERK1/2 phosphorylation induced by morphine in SH-SY5Y cells (Fig. 5c).

Cunha et al. reported that morphine peripheral analgesic effect depended on the activation of PI3Kγ/AKT/nNOS/NO/K_ATP_ signaling pathway [33]; thus, we investigated whether or not AKT was involved in the release of HSP70 induced by morphine in SH-SY5Y cells. First, the incubation of SH-SY5Y cells with morphine (200 μM) induced a rapid and transient activation of AKT phosphorylation, especially in 30 min (Fig. 5d). Second, HSP70 release induced by morphine was abrogated by AKT inhibitor (Fig. 5e). SH-SY5Y cells were pretreated with AKT inhibitor (AZD5363, 2 μM) for 15 min before morphine administration. Third, we investigated whether or not ERK1/2 is downstream to AKT. We found that AKT inhibitor (AZD5363, 2 μM) prevented ERK1/2 phosphorylation induced by morphine in SH-SY5Y cells (Fig. 5f). Moreover, we found that glibenclamide (200 μM) preincubation (15 min) did not affect AKT phosphorylation induced by morphine in SH-SY5Y cells. (Fig. 5g).

**Fig. 6:**
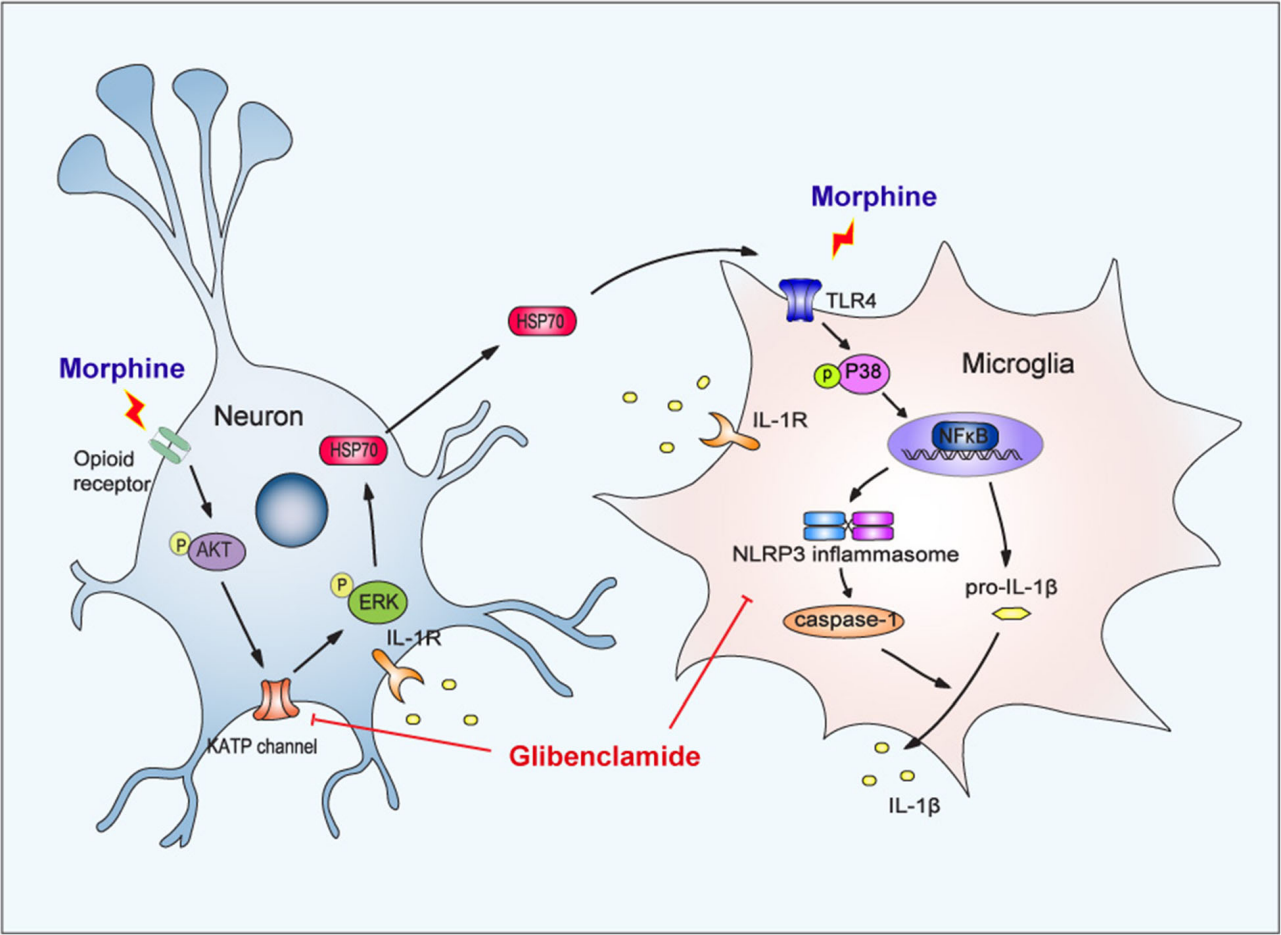
Schematic model indicates that glibenclamide alleviates morphine tolerance by inhibiting HSP70 release and NLRP3 inflammasome activation. Morphine activates TLR4 by inducing the release of HSP70. The secretion of HSP70 is under the control of MOR/AKT/K_ATP_/ERK signal pathway. Extracellular HSP70 induces the activation of microglia and upregulation of neuroinflammatory cytokines including IL-1β via TLR4-NLRP3 inflammasome pathway. Glibenclamide as a K_ATP_ channel blocker inhibits the release of HSP70 induced by morphine and suppresses the activation of NLRP3 inflammasome, consequently alleviating morphine tolerance

At last, we found that morphine-induced release of HSP70 was dependent on μ-opioid receptor. Immunoblot data showed that naloxone (opioid receptor antagonist, 10 μM) inhibited the release of HSP70 induced by morphine (Fig. 5h). Furthermore, naloxone (10 μM) preincubation (15 min) prevented phosphorylation of AKT and ERK1/2 induced by morphine in SH-SY5Y cells (Fig. 6i, j).

## Discussion

In this study, we gave the first evidence that administration of morphine induced the release of HSP70, an endogenous TLR4 ligand, from neurons. Extracellular HSP70 activated microglia and caused inflammatory response, upregulating the level of IL-1β and TNF-α. Morphine promoted the efflux of HSP70 into extracellular environment in a concentration-dependent manner (Fig. 1a). Correspondingly, we found that morphine markedly caused HSP70 to be released into CSF in vivo (Fig. 1f). This result firstly demonstrated that morphine could induce the release of a DAMP, HSP70 and then triggered TLR4-mediated inflammation. The HSP70 in CSF may act as a neuroinflammatory mediator; therefore, we provided a hypothesis that the released HSP70 induced by morphine probably played an important role in neuron-glia crosstalk, especially in neuroinflammation.

Cunha et al. reported that morphine could activate K_ATP_ channel [33]; in our study, pharmacological and molecular evidences supported the involvement of K_ATP_ channel in the regulation of HSP70 release. We demonstrated that glibenclamide, a K_ATP_ channel blocker, could inhibit the release of HSP70 (Fig. 1c, f) increasing intracellular HSP70 upon morphine stimulation in vitro and vivo (Fig. 1d, e). In addition, knockdown of Kir6.2 also suppressed morphine-induced extracellular HSP70 (Fig. 1k).

Moreover, DAMPs including HSP70, could evoke inflammatory response via TLR4 [29]. In our study, we demonstrated that extracellular HSP70 upregulated the phosphorylation of p38 MAPK and NF-κB p65 (Fig. 2a, b) and increased the transcription of IL-1β and TNF-α (Fig. 2c, d). Anti-HSP70 antibody could significantly inhibit the upregulation of IL-1β and TNF-α mRNA level caused by CM collected from SH-SY5Y cells treated by morphine (Fig. 2g, h). TLR4 antagonist, TAK242, abolished the increase of IL-1β and TNF-α caused by HSP70, and interestingly, however, p38 MAPK inhibitor, SB202190, only suppressed IL-1β but not has affected TNF-α (Fig. 2c, d). Park et al. reported that LPS induced TNF-α expression by activating NF-κB via the PKCα/PI3K/AKT/JNK pathway, which was in turn dependent on MyD88/IRAK1 [56], implying that extracellular HSP70 may induce the expression of TNF-α not by p38. In addition, extracellular HSP70 elevated the protein level of NLRP3, which acted as signal 1 for the activation of NLRP3 inflammasome (Fig. 2e). In the presence of ATP, HSP70 induced the activation of caspase-1 and the maturation of IL-1β (Fig. 2f).

These results indicated that HSP70 and K_ATP_ channel played important roles in neuroinflammation and implicated their significance in morphine tolerance. Therefore, we questioned whether anti-HSP70 neutralizing antibody or K_ATP_ channel blocker could improve morphine tolerance. We found that anti-HSP70 neutralizing antibody attenuated morphine tolerance (Fig. 3b). Excitedly, K_ATP_ channel blocker, glibenclamide markedly improved morphine tolerance (Fig. 3a). Moreover, glibenclamide significantly inhibited morphine-induced activation of microglia (Fig. 3c) and the phosphorylation of p38 MAPK and NF-κB p65 in the spinal cord (Fig. 3d, e). Immunofluorescence staining data revealed glibenclamide decreased CGRP (Fig. 3g), which was a peptide released by primary afferents and was able to mediate the activation of NMDA receptors in neurons [57]. Glibenclamide also downregulated c-fos (Fig. 3f), which was implicated in pain transmission and morphine tolerance [58]. It has been reported that glibenclamide has dual pharmacological effects, namely blocking of K_ATP_ channels and of the cystic fibrosis transmembrane conductance regulator (CFTR). In order to further confirm that glibenclamide attenuated morphine tolerance via blocking K_ATP_ channel, we utilized gliquidone, another kind of sulfonylurea K_ATP_ channel blocker, to supply experiments. We found that gliquidone (2 μg/10 μL, i.t.) also attenuated morphine tolerance. Furthermore, gliquidone (200 μM) inhibited the release of HSP70 induced by morphine (200 μM, 12 h) in SH-SY5Y cells (Additional file 7: Figure S7). These evidences suggested that glibenclamide inhibited morphine tolerance via the K_ATP_ channels blockade.

The effect of K_ATP_ channel in neuroinflammation is complicated. Studies reported that classic K_ATP_ channel opener diazoxide inhibited rotenone-induced microglia activation and production of pro-inflammatory cytokines via the stabilization of mitochondrial membrane potential and inhibition of p38/c-Jun-N-terminal kinase (JNK) activation in microglia [59]. Moreover, another K_ATP_ channel opener cromakalim suppressed morphine-induced astrocyte activation by suppressing the JNK pathway, reducing IL-1β, and attenuating morphine tolerance [60].

On the other hand, K_ATP_ channel blocker, glibenclamide, reduced adverse neuroinflammation and behavioral outcomes in CNS injury [61]. Glibenclamide displayed a protective role in inflammation-induced injury in various systems, including cardiology [62], CNS [63–65] and ischemia-reperfusion (IR) injury [65, 66]. Glibenclamide suppressed phosphorylation of NF-κB, ERK 1/2, and JNK in RAW264.7 cells and reduced the release of TNF-α, which alleviated the progression of atherosclerosis in mice [62]. Sun et al. indicated that K_ATP_ channel activation was important in the pathway of IR injury and was a promising target for protecting neurovascular function in stroke [67].

In our study, we chose glibenclamide not only because of its protective role in neurological diseases by inhibiting K_ATP_ channels, but also because that glibenclamide could abolish the activation of NLRP3 inflammasome in bone marrow-derived macrophages (BMDMs) [68], although the exact mechanism was not fully understood. Glibenclamide inhibited NLRP3 inflammasome via upstream inhibition of the inflammasome and downstream blockade of the P2X7 receptor, which then reduced NLRP3 inflammasome-mediated caspase-1 activation and inhibited the maturation of IL-1β [68]. Our results indicated that glibenclamide suppressed NLRP3, caspase-1, and IL-1β in mice spinal cord in morphine tolerance (Fig. 4a–c). Although glibenclamide inhibited morphine-induced activation of NLRP3 inflammasome, it did not sufficiently suppress the level of pro-IL-1β and pro-casp1. We speculate the reasons for that as follows: firstly, in our study, we found that morphine activated TLR4 by inducing the release of HSP70 from neurons to evoke NF-κB activation; however, there were still other classical pathways for morphine to activate TLR4-NF-κB, such as binding with MD-2, a TLR4 accessory protein [4], or triggering TNF-α-mediated activation of NF-κB [69, 70]. According to our study, glibenclamide inhibited the maturation of IL-1β by suppressing signal 2 of NLRP3 inflammasome, and partially inhibited signal 1 for the activation of NLRP3 inflammasome. Therefore, in an in vivo study, glibenclamide did not display obvious inhibitory effects in pro-IL-1β and pro-casp1. Secondly, the activation of NLRP3 inflammasome is a dynamic process. The two steps involved in this process were both affected by morphine. The conversion of pro-caspase1 and pro-IL-1β to their mature forms following their upregulation is induced by morphine. Morphine increased the pool of caspase1 and IL-1β including precursor form and mature form, and glibenclamide inhibited the transcription and maturation of caspase1 and IL-1β, decreasing the pool of them. Therefore, the change of pro-caspase1 and pro-IL-1β were not obvious after the administration of glibenclamide.

Furthermore, in in vitro studies, we found that glibenclamide inhibited caspase-1 activation and the maturation of IL-1β induced by morphine and LPS in the presence of ATP (Fig. 4d). These results suggested that glibenclamide could directly inhibit morphine-induced activation of NLRP3 inflammasome; moreover, our study also demonstrated that glibenclamide blocked the interaction between HSP70 and TLR4 by inhibiting HSP70 release, implying that glibenclamide had a dual inhibition on the activation of NLRP3 inflammasome.

Finally, we explored the mechanism of HSP70 release from neurons caused by morphine. Previous studies indicated that stress-induced secretion of HSP70 was mediated by ERK1/2 [54], and the activation of ERK1/2 played an important role in morphine-induced tolerance [71–73]. Our data showed that phosphorylation of ERK1/2 was significantly elevated after morphine administration (Fig. 5) and SCH772984 (an ERK1/2 inhibitor) inhibited the morphine-induced release of HSP70 (Fig. 5b). Studies reported that H_2_S exerted a protective effect against cerebral hypoxia-induced neuronal cell death via K_ATP_/PKC/ERK1/2/HSP90 pathway [55]; they proved that glibenclamide abolished the effect of NaHS on ERK1/2 phosphorylation in SH-SY5Y cells, indicating that ERK1/2 activation is downstream of K_ATP_ channel; accordingly, we found that glibenclamide (200 μM) prevented ERK1/2 phosphorylation induced by morphine in SH-SY5Y cells (Fig. 5c).

Compelling evidences had accumulated indicating that AKT was important in morphine tolerance [74, 75], and our results showed that morphine induced a rapid phosphorylation of AKT, especially in 30 min (Fig. 5d). Merighi et al. reported that morphine mediated a proinflammatory phenotype via μ-opioid receptor/PKCε/Akt/ERK1/2 signaling pathway [75], which is consistent with our result that AKT inhibitor prevented the phosphorylation of ERK1/2 induced by morphine in SH-SY5Y cells (Fig. 5). Another study performed by Cunha et al. showed that morphine peripheral analgesic effect depended on the activation of PI3Kγ/AKT/nNOS/NO/K_ATP_ signaling pathway [33], indicating that AKT was upstream of K_ATP_ channel, and we found that AKT inhibitor abrogated the release of HSP70 (Fig. 5e). Moreover, we found that glibenclamide did not affect AKT phosphorylation induced by morphine (Fig. 5g). At last, we indicated that morphine-induced release of HSP70 was dependent on μ-opioid receptor. Immunoblot data showed that naloxone inhibited the release of HSP70 and the phosphorylation of AKT and ERK induced by morphine (Fig. 6h–j).

## Conclusion

For the first time, we demonstrated that morphine could induce the release of HSP70 from neurons via MOR/AKT/K_ATP_/ERK pathway, providing an alternative pathway, which mediated morphine-induced activation of TLR4 in microglia. The extracellular HSP70 activated microglia via TLR4 and primed NLRP3 inflammasome as signal 1. Moreover, our study provided a clinically safe and effective hypoglycemic drug, glibenclamide, which inhibited HSP70 release by blocking K_ATP_ channel and suppressed NLRP3 inflammasome activation, which consequently attenuated morphine tolerance. Our findings may represent a bright inspiration or provide a promising target for the treatment of morphine tolerance.

## Additional files

Additional file 1: Figure S1. MTT experiments showed that different concentrations of morphine that did not affect cell proliferation. SH-SY5Y cells were incubated with different concentrations (100, 200, 400 μM) of morphine for 12 h, then cell viability and cytotoxicity were detected by MTT experiment. (*n* = 4) (data were analyzed by one-way ANOVA). (JPEG 81 kb)

Additional file 2: Figure S2. Morphine did not affect the release of HSP70 in microglia. BV-2 cells were incubated with different concentrations (100, 200, 400 μM) of morphine for 12 h, then the supernatants of BV-2 cells were collected and analyzed by western blot. (*n* = 3) (data were analyzed by one-way ANOVA). (JPEG 92 kb)

Additional file 3: Figure S3. Glibenclamide co-administration with morphine improved chronic morphine tolerance in rats. Morphine (10 μg/10 μL) was intrathecally injected with glibenclamide (2 μg/10 μL) once daily, and the MPE was measured 1 h after the first injection of each day. (*n* = 6) (data were analyzed by two-way ANOVA. ***P < 0.001 vs. morphine-treated group). (JPEG 112 kb)

Additional file 4: Figure S4. Glibenclamide co-administration with morphine did not affect acute morphine analgesic effect. Morphine (10 μg/10 μL, i.t.) with or without glibenclamide (0.08, 0.4, and 2 μg/10 μL) were injected into mice and analgesia was assessed at the first day. (*n* = 8) (data were analyzed by two-way ANOVA). (JPEG 124 kb)

Additional file 5: Figure S5. Anti-HSP70 neutralizing antibody did not affect acute morphine analgesic effect. Morphine (10 μg/10 μL, i.t.) with or without anti-HSP70-neutralizing antibody (200 μg/kg, i.t.) were injected into mice and analgesia was assessed at the first day. (*n* = 6) (data were analyzed by two-way ANOVA). (JPEG 118 kb)

Additional file 6: Figure S6. Glibenclamide (2 μg/10 μL) did not affect the blood glucose threshold after 1 h of its administration compared with the vehicle group. Blood samples were collected from the tail vein at the indicated time to measure blood glucose levels by ACCU-CHEK Active blood glucose monitoring system. (*n* = 8) (data were analyzed by two-way ANOVA). (JPEG 97 kb)

Additional file 7: Figure S7. Gliquidone attenuated morphine tolerance and suppressed the release of HSP70 induced by morphine in SH-SY5Y cells. Tail-flick method was performed to evaluate the effect of gliquidone on the morphine tolerance. Data were shown as percentage of MPE (A) Gliquidone co-administration with morphine improved chronic morphine tolerance in mice (*n* = 8). Morphine (10 μg/10 μL) was intrathecally injected with gliquidone (2 μg /10 μL) once daily, and the MPE was measured 1 h after the first injection of each day. (B) Gliquidone administration (200 μM, 15 min) prior to morphine (200 μΜ, 12 h) prevented the morphine-induced HSP70 release in SH-SY5Y cells. Supernatants were collected 12 h after morphine treatment and determined by western blot. (*n* = 3) (A: data were analyzed by two-way ANOVA. B: data were analyzed by one-way ANOVA. A: **P < 0.01, ***P < 0.001 vs. morphine-treated group. B: ***P < 0.001 vs. vehicle, ##*P* < 0.01 vs. morphine-treated group). (JPEG 79 kb)

## Abbreviations

ABC: ATP-binding cassette
ASC: Apoptosis-associated speck-like protein
ATP: Adenosine triphosphate
BMDMs: Bone marrow-derived macrophages
CFTR: Cystic fibrosis transmembrane conductance regulator
CGRP: Calcitonin gene-related peptide
CM: Conditional medium
CSF: Collection of cerebrospinal fluid
DAMP: damage-associated molecular pattern
DAPI: 4′,6-Diamidino-2-phenylindole
DMSO: Dimethyl sulfoxide
ECM: Extracellular matrix
FBS: Fetal bovine serum
FITC: Fluorescein isothiocyanate
HMGB1: High-mobility group box 1
HRP: Horseradish peroxidase
HSP70: Heat shock protein 70
HSPs: Heat shock proteins
i.t.: Intrathecal
Iba1: Ionized calcium-binding adapter molecule-1
IL-1β: Interleukin-1β
IR: Ischemia-reperfusion
JNK: c-Jun-N-terminal kinase
LPS: Lipopolysaccharide
MAPK: Mitogen-activated protein kinase
MOR: μ-Opioid receptor
MPE: Maximal potential effect
NF-κB: Nuclear factor-κB
NLRP3: NOD-like receptor protein 3 inflammasome
PBS: Phosphate-buffered saline
qPCR: Quantitative real-time PCR
RIPA: Radio immunoprecipitation assay
TLR4: Toll-like receptor 4
TNF-α: Tumor necrosis factor-α
