# Supplementary figures and images for: Blocking ATP-sensitive potassium channel alleviates morphine tolerance by inhibiting HSP70-TLR4-NLRP3-mediated neuroinflammation
Source: J Neuroinflammation. 2017 Nov 25;14:228. doi: 10.1186/s12974-017-0997-0 (PMC5702153; doi:10.1186/s12974-017-0997-0)

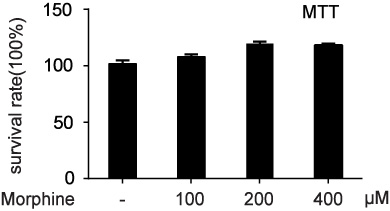

Supplement: Supplementary file 1 — MTT experiments showed that different concentrations of morphine that did not affect cell proliferation. SH-SY5Y cells were incubated with different concentrations (100, 200, 400 μM) of morphine for 12 h, then cell viability and cytotoxicity were detected by MTT experiment. (n = 4) (data were analyzed by one-way ANOVA). (JPEG 81 kb) [file 12974_2017_997_MOESM1_ESM.jpg]

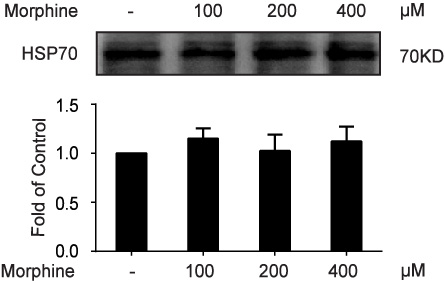

Supplement: Supplementary file 2 — Morphine did not affect the release of HSP70 in microglia. BV-2 cells were incubated with different concentrations (100, 200, 400 μM) of morphine for 12 h, then the supernatants of BV-2 cells were collected and analyzed by western blot. (n = 3) (data were analyzed by one-way ANOVA). (JPEG 92 kb) [file 12974_2017_997_MOESM2_ESM.jpg]

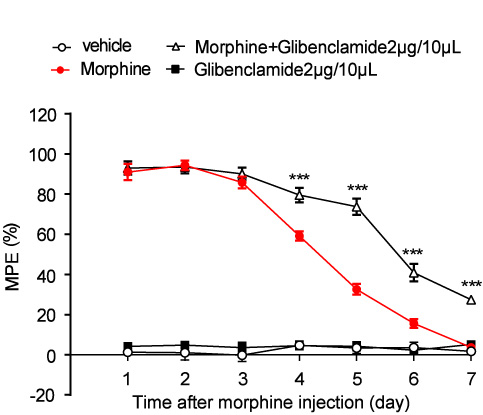

Supplement: Supplementary file 3 — Glibenclamide co-administration with morphine improved chronic morphine tolerance in rats. Morphine (10 μg/10 μL) was intrathecally injected with glibenclamide (2 μg/10 μL) once daily, and the MPE was measured 1 h after the first injection of each day. (n = 6) (data were analyzed by two-way ANOVA. ***P < 0.001 vs. morphine-treated group). (JPEG 112 kb) [file 12974_2017_997_MOESM3_ESM.jpg]

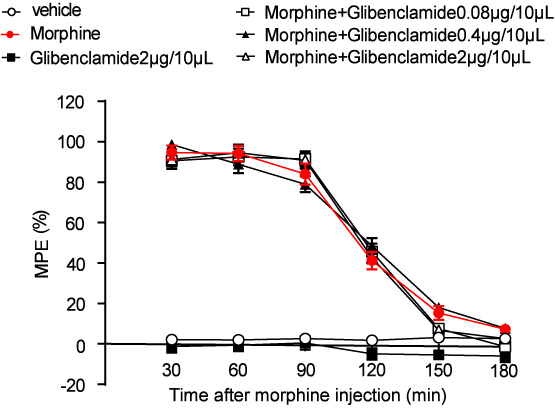

Supplement: Supplementary file 4 — Glibenclamide co-administration with morphine did not affect acute morphine analgesic effect. Morphine (10 μg/10 μL, i.t.) with or without glibenclamide (0.08, 0.4, and 2 μg/10 μL) were injected into mice and analgesia was assessed at the first day. (n = 8) (data were analyzed by two-way ANOVA). (JPEG 124 kb) [file 12974_2017_997_MOESM4_ESM.jpg]

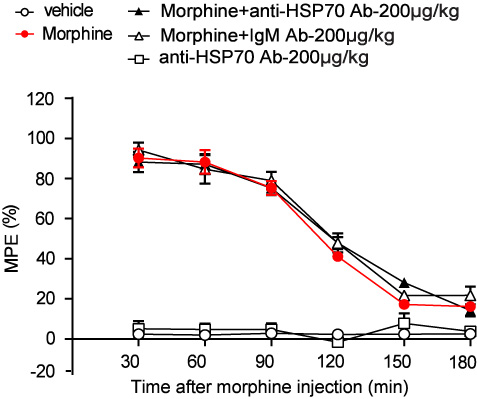

Supplement: Supplementary file 5 — Anti-HSP70 neutralizing antibody did not affect acute morphine analgesic effect. Morphine (10 μg/10 μL, i.t.) with or without anti-HSP70-neutralizing antibody (200 μg/kg, i.t.) were injected into mice and analgesia was assessed at the first day. (n = 6) (data were analyzed by two-way ANOVA). (JPEG 118 kb) [file 12974_2017_997_MOESM5_ESM.jpg]

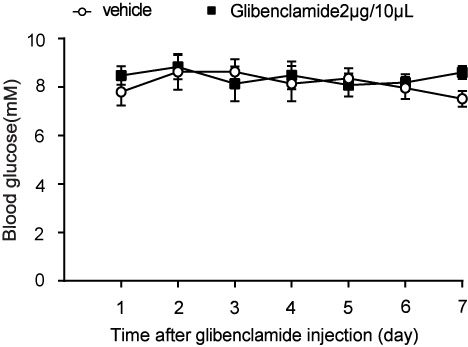

Supplement: Supplementary file 6 — Glibenclamide (2 μg/10 μL) did not affect the blood glucose threshold after 1 h of its administration compared with the vehicle group. Blood samples were collected from the tail vein at the indicated time to measure blood glucose levels by ACCU-CHEK Active blood glucose monitoring system. (n = 8) (data were analyzed by two-way ANOVA). (JPEG 97 kb) [file 12974_2017_997_MOESM6_ESM.jpg]

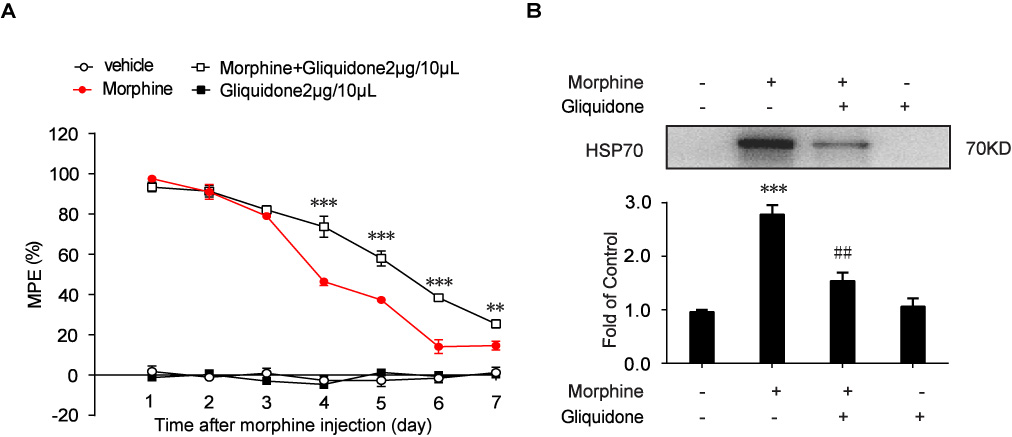

Supplement: Supplementary file 7 — Gliquidone attenuated morphine tolerance and suppressed the release of HSP70 induced by morphine in SH-SY5Y cells. Tail-flick method was performed to evaluate the effect of gliquidone on the morphine tolerance. Data were shown as percentage of MPE (A) Gliquidone co-administration with morphine improved chronic morphine tolerance in mice (n = 8). Morphine (10 μg/10 μL) was intrathecally injected with gliquidone (2 μg /10 μL) once daily, and the MPE was measured 1 h after the first injection of each day. (B) Gliquidone administration (200 μM, 15 min) prior to morphine (200 μΜ, 12 h) prevented the morphine-induced HSP70 release in SH-SY5Y cells. Supernatants were collected 12 h after morphine treatment and determined by western blot. (n = 3) (A: data were analyzed by two-way ANOVA. B: data were analyzed by one-way ANOVA. A: **P < 0.01, ***P < 0.001 vs. morphine-treated group. B: ***P < 0.001 vs. vehicle, ##P < 0.01 vs. morphine-treated group). (JPEG 79 kb) [file 12974_2017_997_MOESM7_ESM.jpg]
